# Using the Injection System as a Sensor to Analyze the State of the Electronic Automotive System

**DOI:** 10.3390/s25185814

**Published:** 2025-09-18

**Authors:** Matej Kucera, Miroslav Gutten, Daniel Korenciak, Michal Prazenica, Tomasz N. Koltunowicz

**Affiliations:** 1 Department of Mechatronics and Electronics, Faculty of Electrical Engineering and Information Technology, University of Zilina, Univerzitna 1, 01026 Zilina, Slovakia; matej.kucera@uniza.sk (M.K.); daniel.korenciak@uniza.sk (D.K.); 2 Department of Electrical Apparatus and High Voltages Technology, Lublin University of Technology, 20-618 Lublin, Poland; t.koltunowicz@pollub.pl

**Keywords:** injection system, diagnostic system, pressure, series resistance, diagnostics

## Abstract

This paper presents a novel diagnostic method that employs the fuel injection system as a sensor for monitoring internal combustion engine pressure, analysing series resistance to detect connector degradation, and evaluating needle movement within the injector’s magnetic core. Experimental results showed that reducing the injector needle opening from 100% to 20% of its maximum displacement caused up to a 35% reduction in peak current amplitude and a 0.2 ms delay in coil charging. Increasing fuel pressure from 0.3 bar to 2.5 bar resulted in a rise in peak current by approximately 35% and an extension of coil charging delay by 0.4 ms. Furthermore, increasing the series resistance from 0.2 Ω to 2.0 Ω reduced the current amplitude by nearly 50% and significantly distorted the waveform, simulating connector oxidation or wear. Comparative analysis of reference and fault current waveforms confirmed that variations in electrical parameters correlate with injector needle displacement, fuel pressure, and resistance changes. Finally, an automated diagnostic system was developed that achieved over 90% accuracy in independently detecting injector faults based on current waveform characteristics.

## 1. Introduction

Various methods are employed to diagnose ignition and injection systems in internal combustion engines, both during engine operation and when the engine is stationary. Stationary testing on dedicated test benches offers precise fault identification; however, it poses practical challenges as it requires taking the vehicle out of service. An alternative is model-based analysis, which assesses whether the observed system behaviour aligns with expected performance or indicates a fault. This approach has gained traction due to modern diagnostic tools that allow automatic fault detection by comparing real-time measurements with model-generated datasets [1,2,3].

Despite advancements, ensuring the reliability of data from automotive sensors remains a challenge, as these sensors may not immediately detect component failures. Tracking performance trends in internal combustion engines and using simulated failure scenarios in ignition and injection systems can enhance fault analysis and maintenance planning. A distinctive diagnostic method involves combining the analysis of secondary voltage waveforms from spark plugs with time-resolved current measurements from fuel injectors. By evaluating historical waveform data, it becomes possible to determine whether a failure originates from an electrical issue in the ignition system, spark plug degradation, an injection fault, or an engine performance problem. Within this diagnostic context, the spark plug or injector can effectively function as a sensor for the engine’s fuel system [4,5].

Developing a diagnostic model requires constructing a mechanical representation of real-world processes, as demonstrated by Hung et al. [6] in their analysis of injection systems. Previous simulations have investigated how the size and position of ferromagnetic components influence magnetic flux, air gaps, and input voltage characteristics [7]. Czarnigowski et al. [8] emphasized that electric current waveforms serve as an effective tool for comparing injector performance, offering a precise method for verification. Their experimental results also explored how variations in supply pressure and voltage affect the injector opening time.

Mathematical models that describe the dynamic behavior of fuel injectors during operation have been shown to enable precise determination of the onset and duration of injection events. These models also provide a foundation for the development of advanced diagnostic methods suitable for integration into modern engine control units [9,10]. Similar approaches have been applied to ignition systems of spark-ignition engines, where two-port network parameters were employed to analyze voltages and currents at system terminals [11]. In parallel, several diagnostic tools have been proposed for the identification of injector malfunctions and misfires [12,13].

Such studies have led to injector models capable of predicting opening delays and injection durations as functions of fuel pressure and supply voltage. Furthermore, diagnostic strategies based on voltage and current waveform analysis in both injection and ignition systems have been used to evaluate combustion efficiency, detect misfires, and assess overall combustion conditions. By applying advanced mathematical techniques to waveform comparison and incorporating degradation factors, the injection system itself can effectively function as a sensor, thereby offering deeper insight into the processes occurring within the combustion chamber.

To support these developments, physical models of injection systems have been constructed to simulate real operating conditions and to explore different fault scenarios. However, limited attention has been given to how specific fault mechanisms—such as partial needle sticking, variations in fuel pressure, or increased series resistance caused by connector wear and oxidation—affect the electrical characteristics of injectors. Addressing this gap, the present work contributes a systematic analysis of these effects on injector current waveforms, establishing their potential as diagnostic indicators of early-stage injector degradation. In addition to waveform analysis, the study integrates non-contact diagnostic techniques, including electromagnetic, thermal imaging, and acoustic field methods, to provide a comprehensive framework for injector condition monitoring under realistic operational conditions.

## 2. Theoretical Evaluation of the Injection System Functioning as a Sensor for Monitoring Processes Within an Internal Combustion Engine

The electronic fuel injector is a vital component of the gasoline engine’s fuel system, operating as a solenoid valve. The motion of its active core is influenced by friction, spring force, and fuel pressure, causing the rising current curve to deviate slightly from that of an ideal inductor [14].

Changes in magnetic permeability are directly affected by the movement of the needle valve. The injector’s opening phase is reflected in the current waveform, while its closing is indicated by the voltage characteristics. Any partial or complete obstruction of the injector’s moving elements is considered a malfunction, as detected through the measured signal characteristics [15].

### 2.1. Operating Principle of the Fuel Electronic Injector

**Figure 1:**
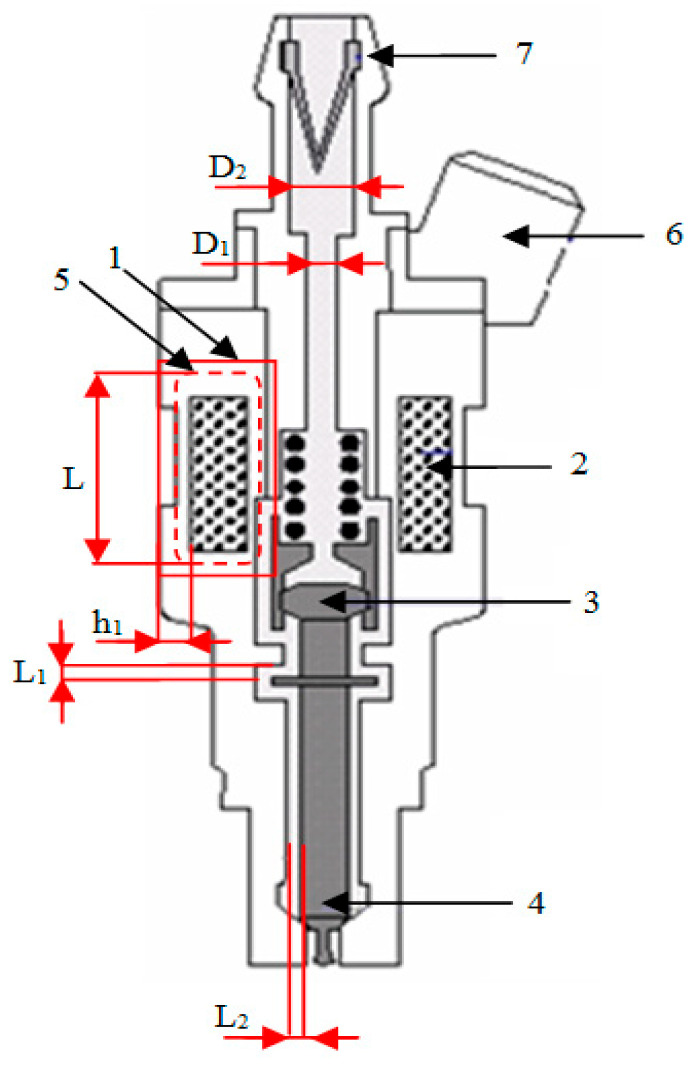
Structure of an electronic fuel injector: 1—core, 2—coil, 3—armature, 4—needle valve, 5—magnetic yoke, 6—electrical terminal, 7—fuel filter [18].

The electronic fuel injector functions as an electromagnetic valve, with its internal structure illustrated in Figure 1. Its main components include the armature, needle valve, iron core, electromagnetic coil, and return spring. Prior to the injection process, the needle valve is held tightly against the injector seat by the combined force of the return spring and fuel pressure.

When electrical current flows through the electromagnetic coil, the resulting magnetic force overcomes the opposing forces of the spring, fuel pressure, and friction. This causes the needle valve to lift, allowing fuel to be sprayed into the combustion chamber. Once the current supply to the coil is interrupted, the magnetic force quickly dissipates, and the return spring forces the needle valve back into its closed position, thus completing the injection cycle [16,17].

### 2.2. Magnetic Field Evaluation of the Electronic Fuel Injector’s Core Structure

**Figure 2:**
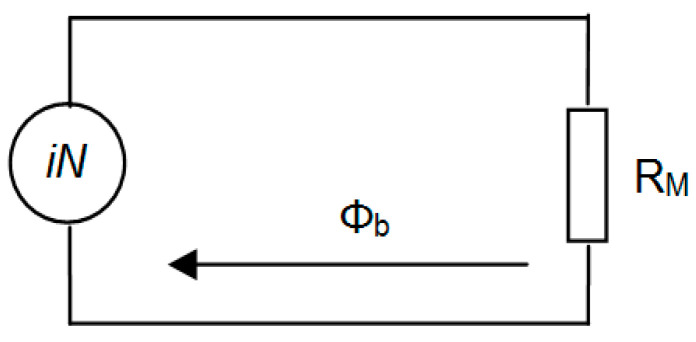
Injector equivalent magnetic circuit [15,18].

The equivalent magnetic circuit of the electronic fuel injector is illustrated in Figure 2. Here, *i.N* represents the magnetomotive force (with *N* denoting the number of coil turns), *Φ*_b_ stands for the magnetic flux, and *R*_M_ indicates the total magnetic reluctance of the magnetic path. Based on Maxwell’s equations, the electromagnetic force acting across the working air gap *L*_1_ can be defined accordingly

(1) $$F=\frac{\emptyset_{b}^{2}}{2\mu_{0}S}$$

where *μ*_0_ is vacuum magnetic permeability, and *S* is cross-sectional area of air gap.

Assuming that the outer and inner diameters of the iron core are designated *D*_2_ and *D*_1_ as shown in Figure 1, then

(2) $$S=\frac{\pi \left({D_{2}}^{2}-{D_{1}}^{2}\right)}{4}$$

Neglecting the effects of the propagated magnetic flux as well as the working air gap *L*_1_ and the sliding air gap *L_2_* according to Figure 1, the magnetic reluctance can be expressed as

(3) $$R_{ml_{1}}=\frac{L_{1}}{\mu_{0}S}$$

(4) $$R_{ml_{2}}=\frac{1}{{2\pi \mu }_{0}h_{1}}ln\frac{D_{2}+2L_{2}}{D_{2}}$$

Compared to other components, the cross-sectional area of the air gap in the iron core is relatively small. To simplify the magnetic circuit model, only the magnetic reluctance of the iron core under magnetic pressure is considered. Assuming the length of the iron core is *L*_0_, the magnetic reluctance of the iron core is given by

(5) $$R_{ml_{0}}=\frac{L_{0}}{\mu S}$$

where *μ* represents the magnetic permeability, *H* denotes the magnetic field intensity, and *B* refers to the magnetic flux density.

Ignoring magnetic flux leakage, according to Ampere circuit law

(6) $$\emptyset_{b}\cdot R_{M}=i.N$$

where *R*_M_ = *R*_ml1_ + *R*_ml2_ + *R*_ml0_ [15,18].

### 2.3. Electronic Fuel Injector’s Circuit Analysis

**Figure 3:**
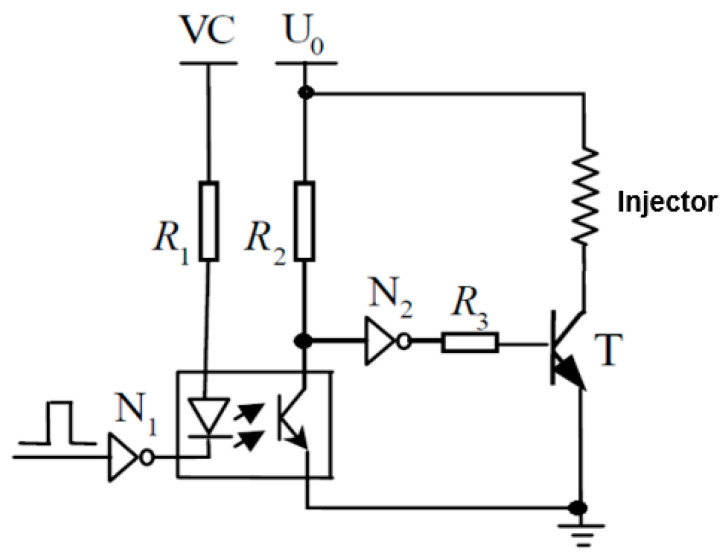
Injector driving circuit [18].

Figure 3 shows a schematic diagram of the electrical circuit that controls the operation of an electronic fuel injector. In its analysis, the saturation voltage of the power transistor in the closed state (i.e., when it is turned on) is neglected for simplicity. The electrical properties of the injector’s electromagnetic coil are represented by the equivalent resistance (*R*) and inductance (*L*). The voltage balance in this circuit can be described by the corresponding equation.

(7) $$U_{0}=R\cdot i+N\frac{{d\emptyset }_{b}}{dt}$$

where *R* includes resistors *R*_1_ + *R*_2_ + *R*_3_ according to Figure 3 [18].

## 3. Injector System Analysis by Time Method

To verify the proper functioning of the injection system, voltage and current time waveform measurements can be combined with non-contact acoustic diagnostics. By analyzing these waveforms, the technical condition of the system can be assessed, and potential faults or performance degradation can be identified. For this purpose, a specialized device has been developed to record the necessary signals. Additionally, this device is capable of simulating fault conditions, allowing observation of how the system’s output characteristics change under such scenarios. Through detailed analysis of these measurements, it becomes possible to evaluate the condition of the injection system, including any decline in its performance.

### 3.1. Description of the Test Equipment

**Figure 4:**
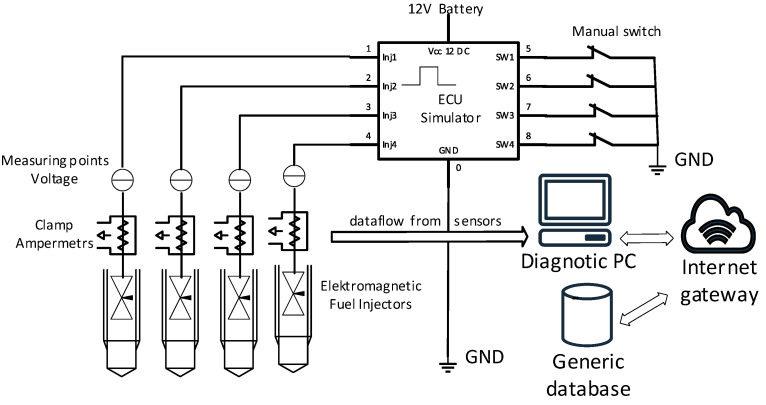
Injector system test equipment wiring diagram.

Figure 4 shows the block diagram of the measurement system for gasoline injectors. The central component of the electronic control unit is a programmable controller, which manages the opening and closing of the transistors. The injectors are connected to the collectors of these transistors. A five-button keypad allows adjustment of the transistor opening duration, while an LCD display indicates the current operating mode.

The test fixture consisted of the Texas Instruments Launchpad F280049C (Dallas, TX, USA) control electronics and the switching part, which is made of optocouplers and Mosfet transistors. The Launchpad is used to simulate pulse width modulation (PWM) and signal fictitious engine speeds. The mounting of the ramp and injectors is solved using custom-made 3D models. The 3D mounting models are designed so that any injection ramp can be tested.

To evaluate the functionality of the injection system and monitor the required signals, a specialized test apparatus was developed for controlling the intake valves of injectors in a spark-ignition engine. The electronic control unit functions similarly to a signal generator, simulating the signals typically produced by an engine control unit (ECU).

**Figure 5:**
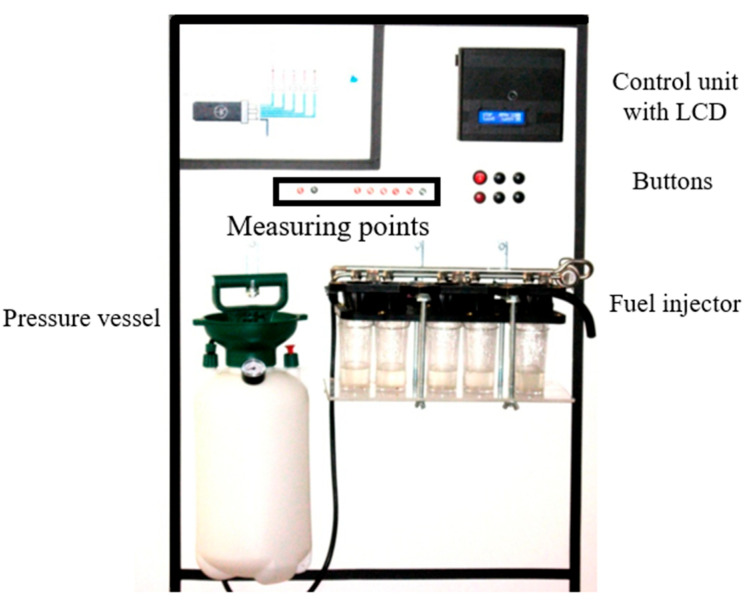
Testing device.

A pressure vessel is used to replicate the function of a fuel pump by generating liquid pressure in the range of 0 to 300 kPa (Figure 5), corresponding to standard fuel pressures in vehicles. Fuel from this vessel is directed into a common manifold that supplies all connected injectors.

The ranges of selected pressures were chosen based on typical values achieved in normal operation during steady-state engine modes, with a focus on comparability and repeatability of experiments in laboratory conditions.

Beneath each injector, calibrated measuring cylinders are placed to collect the injected fuel. This setup enables a direct comparison of the fuel volume delivered by each injector, allowing for the assessment of their individual flow rates.

Measurements of current and voltage help identify deviations from ideal injector performance. Variations in inductance caused by the movement of the injector’s internal core directly affect the observed electrical characteristics.

### 3.2. Theoretical Analysis of Voltage and Current Characteristics the Fuel Injector

**Figure 6:**
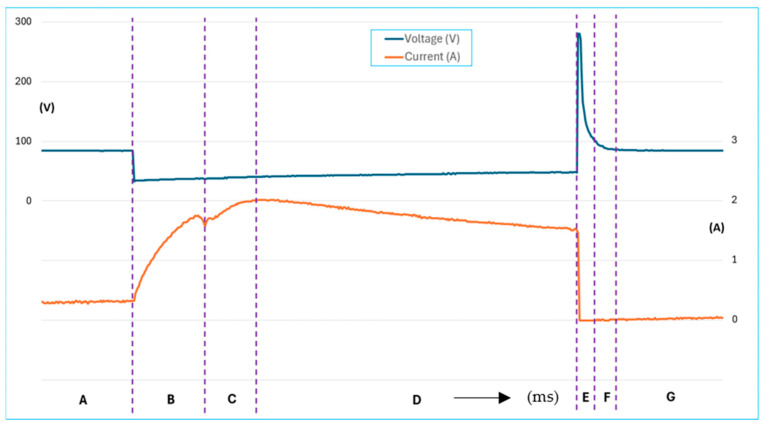
Characteristics of voltage the ignition system and current of fuel injector.

According to Figure 6, Interval A represents the period during which the injector remains inactive. Interval B captures the initial movement of the injector core—after voltage is applied to the coil, the current begins to rise sharply. Interval C marks the start of fuel injection, indicated by a continued increase in current. Interval D corresponds to the phase in which the injector is fully open and operating normally. Once the injection pulse ends, the current drops sharply as the energy stored in the coil is released. When the electromagnetic force can no longer counteract the return spring, the needle valve begins to close. Interval E shows the overvoltage spike caused by the inductive effect as the core returns to its original position. Interval F illustrates the gradual decay of this overvoltage, and Interval G signifies the injector returning to its idle state [17,18].

The typical opening time for an injector with a resistance of 12–16 ohms is approximately 1.5 milliseconds. This duration is influenced by several factors, including fuel pressure, return spring force, core inertia, electromagnetic coil characteristics, and the materials used in the injector’s core and body. In comparison, the closing time is about half of the opening time, as it is governed solely by the return spring and fuel pressure.

**Figure 7:**
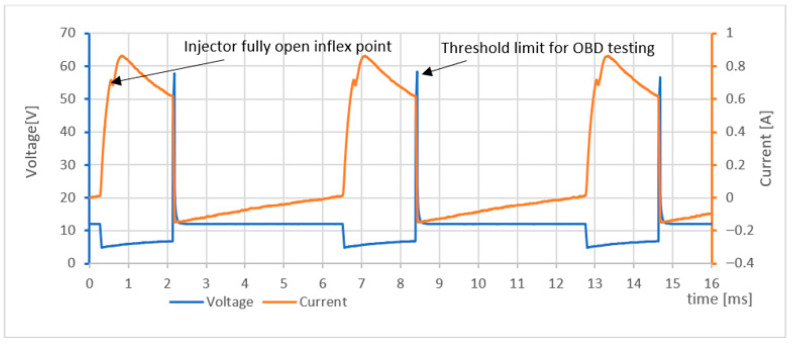
Waveform generated during the operation of the solenoid injector at MPI engine 800 rpm.

Waveform analysis of a fuel injector is a powerful diagnostic method for detecting faults and understanding the behavior of fuel injection systems. It can reveal a wide range of issues, including clogged injectors, electrical malfunctions, and mechanical wear. Figure 7 presents a structured approach to implementing and modeling waveform analysis for effective diagnostics.

**Figure 8:**
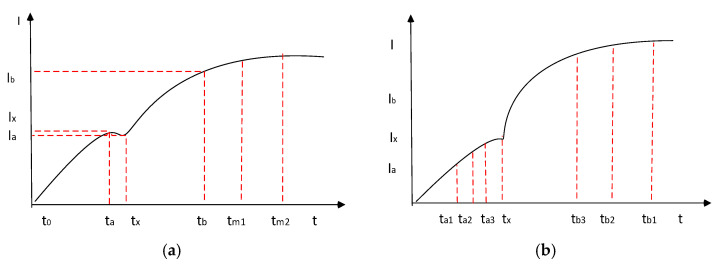
Theoretical analysis of the shape of the injector flow curve. (**a**) depicts idealized current flow during injector operation along with key sampling points that should be recorded when diagnosing the condition of the injection system; (**b**) shows the time profile of the current during the movement of the injector needle valve, measured using a logarithmic scale at seven time intervals.

Figure 8a illustrates an idealized current flow during injector operation, along with the key sampling points that should be recorded when diagnosing the condition of the injection system. By measuring the time labelled *t***_x_**, the duration of injector opening can be evaluated, which depends on both the pressure of the injected fuel and the design of the injector itself. Analysing this time period can help identify issues such as low fuel pressure, a damaged return spring, or delayed needle venting.

By recording the current at the moments *t*_m1_ and *t*_m2_, the peak current value can be determined. Changes in this value may indicate faults in the injector winding—such as a short circuit—which can affect the total series resistance of the coil.

To achieve maximum measurement efficiency, it is essential to minimize the number of samples of the tested signal while still capturing as much relevant information as possible. The *t*_x_ point can be determined experimentally; during its recording, fuel pressure should be maintained within the range of 250 to 300 kPa, and the supply voltage should be between 13.5 and 14.4 V.

Figure 8b presents the time profile of current during the movement of the injector needle valve, measured using a logarithmic scale across seven-time intervals. This measurement method is particularly well-suited for identifying changes in the *t***_x_** time.

**Figure 9:**
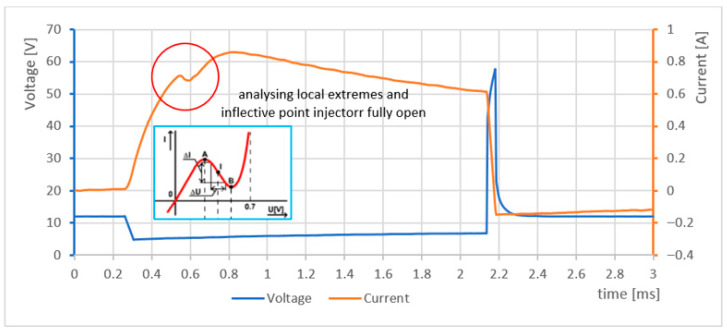
Analysis of local extrema and inflection points in a fully open injector system.

By analysing the recorded time waveforms, one can observe how the movement of the injector needle valve influences inductance, which in turn affects the measured time characteristics. Figure 9 shows the current and voltage waveforms of a fuel injector operating at an idle speed of 716 rpm. Approximately 0.6 ms after the corresponding transistor is switched on, the injector reaches full opening.

A detailed analysis of the waveform segment between 0.5 ms and 0.8 ms, corresponding to the needle valve opening, allows for the determination of key injector parameters and condition. This information can be used to diagnose issues such as low fuel pressure, damage to the injector return spring, or hesitation or blockage in needle valve movement.

### 3.3. Methodological Procedure

For a clearer presentation of the issue, the overall analysis proposal in the article is processed in Table 1. When diagnosing an MPI system, it is first of all essential to develop a relevant methodology proposal, which would ideally allow the results to be verified through a summary of measurements. The implementation of the proposed methodology from a theoretical point of view consists of measuring injectors of N-number of vehicles with the same type of combustion engine, but with different runs under specific conditions during the measurements. The chosen methodology allows us to theoretically and empirically verify the possibility of the influence of injector depreciation on the resulting shape of the current waveform.

Data records obtained using the PicoScope 2205A (Pico Technology, Ltd., Cambridgeshire, UK) oscilloscope were exported to CSV format for analysis. The measurement methodology was applied consistently for all tested vehicles. 10 injection cycle periods were recorded for each injector. Given the four-cylinder configuration of the 1.8t engine, the complete dataset for one vehicle represented a total of 40 current characteristics (10 periods × 4 injectors).

In the first phase, it was necessary to perform time synchronization of the waveforms based on the reference point, which was the beginning of the control pulse (voltage drop to zero accompanied by an increase in current). After synchronization of ten waveforms, averaging was followed by calculating the arithmetic mean value, which eliminated random noise and obtained a representative characteristic of the injector.

In the next phase, a comparative analysis of the four averaged characteristics belonging to individual injectors followed. In the case of mutual overlap, these waveforms were averaged into one characteristic for the given vehicle.

As a result of the above procedure, a normalized current characteristic representing the technical condition of the injection system of the measured vehicle was obtained for each vehicle. This methodology was uniformly applied to all the examined vehicles. Subsequent comparative analysis of these characteristics allowed the identification of differences in the parameters of individual waveforms.

Table 1 proposes a clear solution to the diagnostic problem in Section 3.4 using current dependence with possible technical influences on injector operation and identification of parameter resolution.

**Table 1:** Solving a diagnostic problem using current dependence with technical influences on injector operation and identifying parameter resolution.

Diagnostic Problem	Figure	Conditions	Time/Current Resolution
Comparison of open and blocked valve on injector.	Figure 10	At 0 and 100% injector valve opening.	0.2 ms–1 s
Comparison of open and partially blocked valve on injector.	Figure 11	At 0, 20, 30, 50, 80 and 100% injector valve opening.	0.3 ms–0.5 ms 0.3–0.5 A
Comparison of injector fuel flow with and without pressure.	Figure 12	With and without 2.5 bar pressure.	0.05 ms–1.6 s
Analysis of flow as a function of time at different injector needle openings.	Figure 13	With 0.3, 0.5, 1.5, 2 and 2.5 bar pressure.	1 s–1.7 s 0.37–0.52 A
Comparison of characteristics as a function of injector total series resistance.	Figure 14	At different values of total series resistance.	1 s–1.7 s 0.37–0.52 A

**Figure 10:**
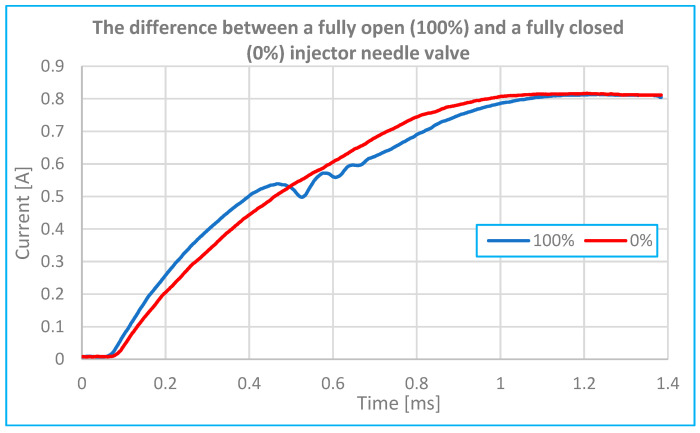
Injector reference current and needle valve stop current.

**Figure 11:**
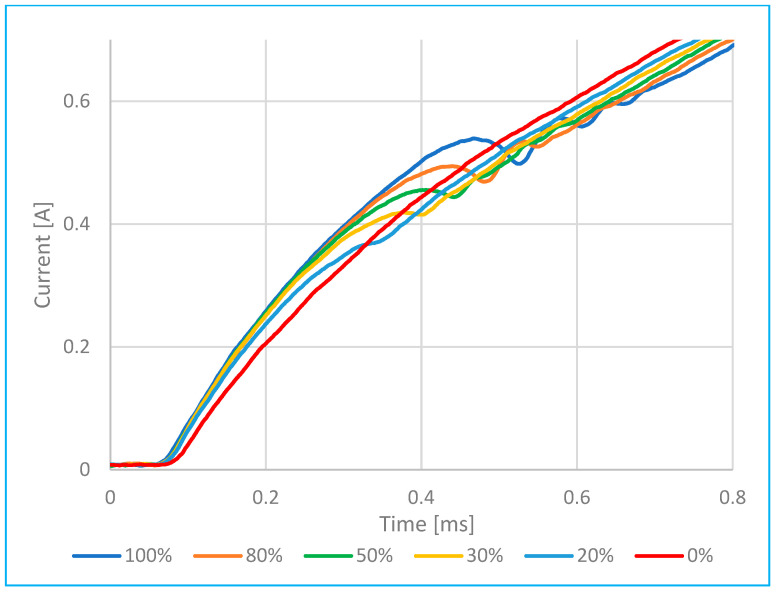
Analysis of the flow rate as a function of time at different injector needle openings (100–80–50–30–20–0%).

**Figure 12:**
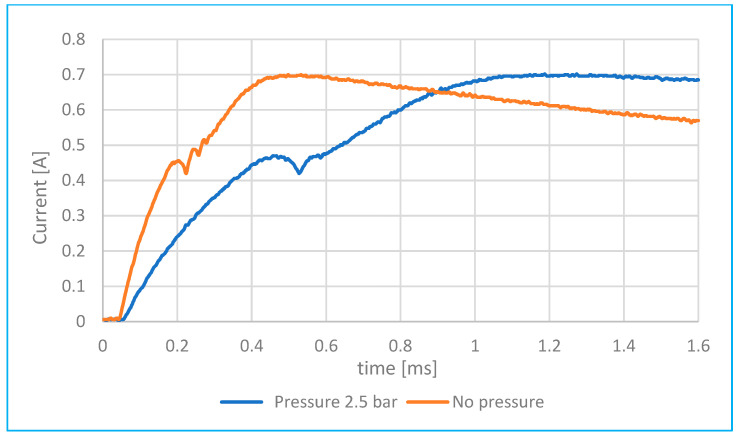
Comparison of fuel injector flow rate versus pressure with and without 2.5 bar pressure.

**Figure 13:**
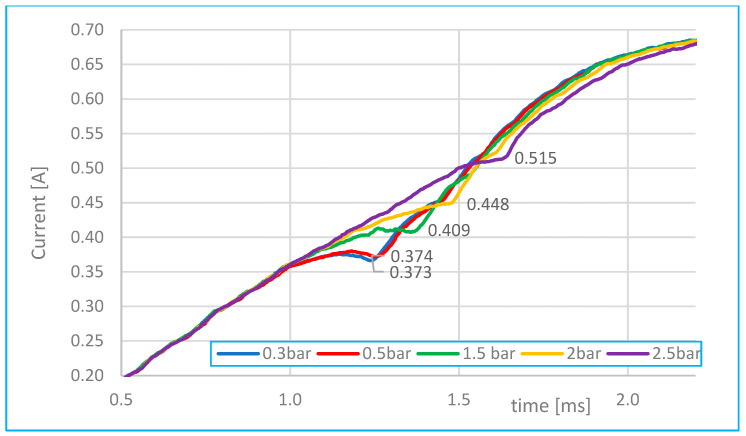
Comparison of characteristics depending on the electric current of the injector and the pressure.

**Figure 14:**
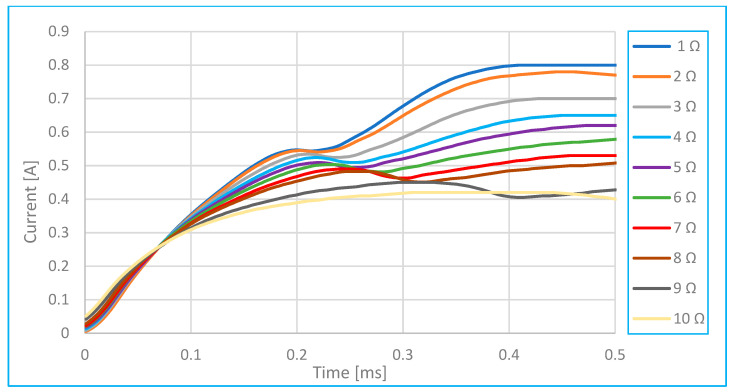
Comparison of characteristics depending on the injector electric current and series resistance.

### 3.4. Experimental Analysis

Analysing fuel injector operation—particularly through time-based measurements of voltage and current—enables a detailed assessment of both its electrical characteristics (e.g., current consumption, rise and fall times) and mechanical behaviour (e.g., needle valve opening and closing dynamics).

One of the most critical factors influencing injector performance, and one that must be considered during analysis, is fuel supply pressure. Deviations from the optimal pressure—either too high or too low—can significantly affect the needle valve’s opening time, which directly influences the quantity of fuel injected. At low pressure, the needle valve may respond more slowly or fail to open fully, resulting in incomplete injection. In contrast, excessive pressure can shorten the opening time or accelerate mechanical wear.

Fuel pressure also influences electrical behaviour during injector operation. For instance, the coil current profile may change depending on the force required to open the valve against varying fuel pressures.

Over time, injector wear or damage will cause deviations in these parameters, leading to changes in the amount of fuel delivered. Figure 10 presents a comparison of the measured characteristics of a reference current and a fault current. The reference current waveform is based on five measurements taken with fuel at 2.5 bar and the needle valve fully open. The oscilloscope sampling rate was set to 100 kS/s, corresponding to a 10 μs interval between samples. The injection duration was set to 5 ms. The fault current waveform was obtained by mechanically locking the needle valve in the open position (0% opening movement).

Signal integrity was verified with a signal-to-noise ratio exceeding 50 dB at critical inputs. Timing measurements were performed with a resolution of 1 µs and an estimated timing error margin of less than ±100 µs, ensuring accurate detection of injector events with a crankshaft angle deviation of less than 1° at engine speeds up to 6000 rpm.

Figure 11 presents the current waveforms over time for various needle valve opening levels—specifically 100–80–50–30–20–0% of the total possible displacement of the injector needle. These represent conditions where fuel injection is either incomplete or in a transient state due to partial needle movement. Figure 14 (in the Discussion section) illustrates the relationship between the displacement of the moving injector needle and the injector opening time.

The pressure in the combustion chamber does not directly influence the injector’s electrical current; however, it does indirectly affect the current waveform through its impact on the movement of the injector needle.

Higher fuel pressure creates greater resistance against the needle, requiring the electromagnetic coil to exert more force to initiate needle movement. When the needle remains stationary longer due to this increased pressure, the inductance of the coil stays lower for a longer period, allowing the current to rise more rapidly at the beginning. Once the needle begins to move and the air gap decreases, the inductance increases, which slows the rate of current rise [5].

Figure 12 compares current waveforms measured at a fuel pressure of 2.5 bar and at zero pressure. The oscilloscope sampling rate was set to 100 kS/s (with a 10 μs interval between samples), and the injection duration was set to 5 ms by the control circuit. At 2.5 bar, the electromagnet must overcome both the return spring force and the fuel backpressure. During this delay, the coil continues to charge, meaning—according to Figure 12—the current takes longer to initiate needle movement.

This delay affects inductance behaviour: as the needle begins to move, the inductance increases, which in turn slows the rate of current rise, resulting in a shallower slope in the current waveform (starting around 0.55 ms, as seen in Figure 12).

Figure 13 illustrates the relationship between electric current and fuel pressure. As fuel pressure increases, the current magnitude also increases, along with a longer coil charging delay. This results in a longer time before the injector needle begins to move.

Series resistance—such as the resistance of the injector coil winding or the wiring in the circuit—has a significant impact on the time profile of the injector current. Typically, this resistance ranges from 0.2 to 2 ohms, depending on the injector type, but it can increase due to damage, oxidation, or connector wear.

As shown in Figure 14, increasing the series resistance causes the current waveform to rise more slowly. The point at which the slope changes—indicating needle movement—shifts to a later time, and the peak current decreases as resistance increases (up to 10 ohms).

## 4. Discussion

Based on Figure 10 and Figure 11, it can be concluded that the movement of the injector needle causes a change in coil inductance—specifically, a decrease—which leads to a faster rise in current at a certain point in time. When the needle (core) is far from the magnetic core (e.g., when the valve is closed), the air gap is large and the inductance is low. As the needle moves closer to the magnetic core, the air gap decreases and the inductance increases.

This shift in inductance is clearly visible on the current–time waveform as a distinct inflection point (or “fold”) and represents a critical diagnostic indicator of injector function.

**Figure 15:**
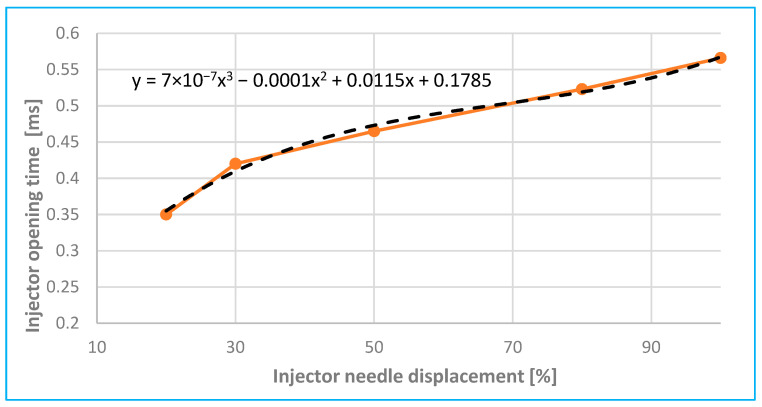
Analysis of injector needle displacement depending on injector opening time.

Figure 15 shows the simulated displacement characteristics of the moving injector needle as a function of the injector opening time (referred to as *t*_x_ in Figure 8b).

If this inflection point is absent or occurs at an atypical time, it may indicate a mechanical or hydraulic fault within the injector. A smaller-than-expected needle opening results in lower pressure and reduced fuel velocity, which impairs fuel atomization. This leads to larger fuel droplets, poorer air–fuel mixing, and ultimately incomplete combustion, contributing to increased soot formation and elevated emissions (e.g., CO, HC).

According to Figure 11 and Figure 15, experimental results showed that reducing the injector needle opening from 100% to 20% of its maximum displacement resulted in a reduction in the peak current amplitude by up to 35%, accompanied by a coil charging delay of 0.20 ms. Compared to [2,6] and [7,8], these are common delay values (maximum up to 0.6 to 1 ms), which also depend on the type and model of injector in question.

**Figure 16:**
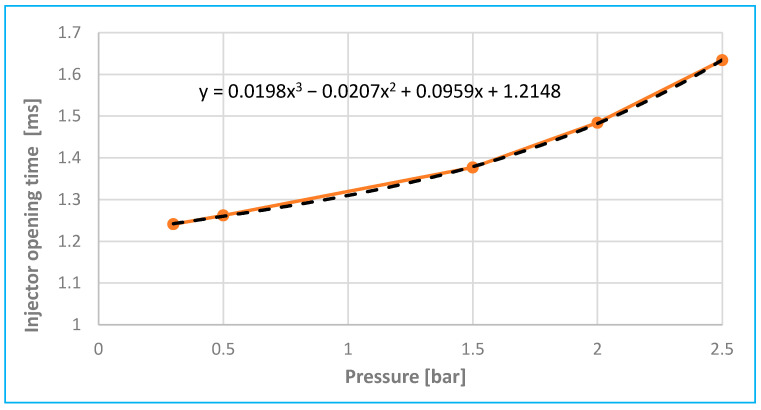
Analysis of pressure depending on injector opening time.

As the fuel pressure gradually increases—up to 2.5 bar as shown in Figure 16 (based on Figure 13)—the solenoid must exert greater force to overcome the opposing back pressure that resists needle movement. As a result, it takes longer for the current to reach a level sufficient to initiate needle displacement, leading to a delayed inflection point in the current waveform.

According to Figure 13 and Figure 16, increasing fuel pressure from 0.3 bar to 2.5 bar resulted in a rise in peak current by approximately 35% and an extension of coil charging delay by 0.4 ms. Compared to [2], these are common delay values (maximum up to 1 ms), which also depend on the type of injector model in question. During tests of fuel injectors of internal combustion engines from a pressure of 0.4 MPa (4 bar) to a pressure of 0.2 MPa (2 bar), it was evident, according to [19], that the change in fuel pressure led to a decrease in the current intensity at the needle lift point of the order of several tens of milliamperes.

**Figure 17:**
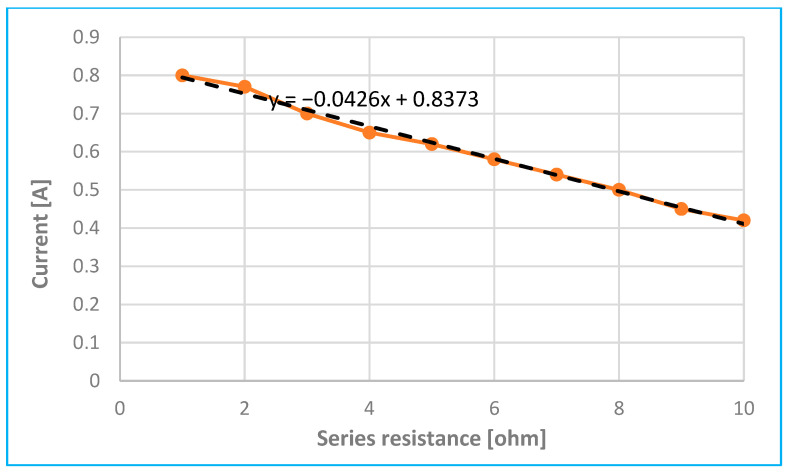
Analysis of current drop due to increase in injector series resistance.

Figure 14 clearly illustrates the effect of changing series resistance on the maximum achievable injector current. Figure 17 further demonstrates this relationship. An increase in series resistance—caused, for example, by partial damage, oxidation, or wear of the injector connectors—can lead to a noticeable reduction in current. Therefore, it is recommended to monitor any decrease in current as a potential indicator of such issues.

Also, according to [2,3,5], a series resistance on the injector in the range of 0.5–2 Ω can significantly affect the current flow and cause a measurable shift in the rise and decrease in the peak amplitude, as shown in Figure 17. According to Figure 14 and subsequently 17, increasing the series resistance from 0.2 Ω to 2.0 Ω reduced the current amplitude by almost 50% and significantly distorted the waveform, simulating connector oxidation or wear.

In addition to a lower maximum current, increased series resistance is also indicated by a slower current rise—the current curve rises more gradually, as shown in Figure 14. The inflection point at time *t*_x_ either shifts to a later time or disappears entirely (indicating that the needle may not move). Because the curve shape and maximum current magnitude change very little with variations in fuel pressure, it is possible to differentiate faults caused by increased series resistance from those caused by fuel pressure issues.

Similarly, the experimental measurement was repeated approximately 20 times with the same or similar type of injector, where the measurement of the time course of the current showed approximately similar results and the same conclusions in all three cases.

All these criteria can be integrated into the proposed automated diagnostic system, enabling it to independently identify potential faults based on the measured current characteristics. The key advantage of the built-in test system illustrated in Figure 4 is the ability to develop a comprehensive database of reference and fault-state measurements for various types of injection systems, allowing for direct comparison and analysis against data from actual vehicle injection systems.

**Figure 18:**
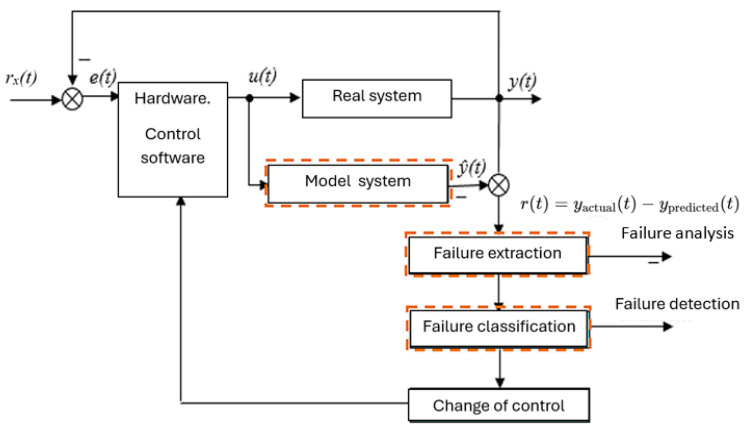
Block diagram of the analysis of failure states of the model system and comparison with the real injection system.

The device can simulate fault conditions in individual components (as shown in Figure 18), which affect electrical parameters that can then be measured and evaluated. The dashed boxes are virtual blocks for analyzing and processing the measured data of the injection system. Using the data in Figure 18, the simulated waveforms and values can be compared with measurements from real systems, facilitating the detection of possible faults in the injection systems of vehicles.

Coil aging, fuel contamination or other problems were not included in the fault analysis, as they would be too complicated problems that cannot be reconciled when identifying the anomaly unambiguously. Therefore, only the most common possible faults that have a major impact on the current flow in the injection system were included in the analysis.

## 5. Conclusions

Injector diagnostics based on current waveform analysis provide an effective method for evaluating their functional condition. The shape of the current during injector activation reflects both the electrical and mechanical characteristics of the system. Increased series resistance is indicated by a slower current rise and a lower peak value. Higher fuel pressure causes a delay in needle lift, resulting in a shift in the characteristic inflection point in the current curve. Mechanical faults, such as a stuck needle, are manifested by the absence of significant changes in the current waveform during activation.

Accurate interpretation of these features allows for precise identification of specific fault types. Comparing measured waveforms with reference signals from fully functional injectors greatly enhances diagnostic accuracy. Additionally, current measurement enables early detection of partial faults that might otherwise only become apparent later.

This diagnostic approach is suitable for both preventive maintenance and precise fault localization within the injection system. Regular analysis of current waveforms can therefore significantly improve the reliability and efficiency of internal combustion engines.

It is necessary to realize the importance of model validation and parameter identification for practical applications. Therefore, in future studies or follow-up work, the inclusion of simulation calculations according to at least Equations (1)–(7) will be considered as potential approaches to comparing model parameters with experimental measurements, and we outline steps that could be taken to adapt the parameters to the validation of the physical model.

These analysis methods can be used for different vehicle models or injection systems, and for the correct system, only a formula with limit values at important points is needed. They are also suitable for preventive maintenance, as well as for the precise localization of faults in the injection system. A regular analysis of the flow path can therefore significantly increase the reliability and efficiency of internal combustion engines, regardless of real-time performance, computing resource requirements and compatibility.

## Data Availability

The original contributions presented in this study are included in the article. Further inquiries can be directed to the corresponding authors.

## References

[1] Ismail M.A.A., Wiedemann S., Bosch C., Stuckmann C. (2021). Design and Evaluation of Fault-Tolerant Electro-Mechanical Actuators for Flight Controls of Unmanned Aerial Vehicles. Actuators.

[2] Maczak J., Wieclawski K., Szczurowski K. (2023). New approach of model based detection of early stages of fuel injector failures. Maint. Reliab..

[3] Li S., Nehl T., Gopalakrishnan S., Omekanda A., Namuduri C., Prasad R.A. Dynamic Solenoid Model for Fuel Injectors. Proceedings of the IEEE Energy Conversion Congress and Exposition.

[4] Papadopoulos P.M., Lymperopoulos G., Polycarpou M.M., Ioannou P. (2022). Distributed Diagnosis of Sensor and Actuator Faults in Air Handling Units in Multi-Zone Buildings: A Model-Based Approach. Energy Build..

[5] Wieclawski K., Maczak J., Szczurowski K. (2020). Electric Current Waveform of the Injector as a Source of Diagnostic Information. Sensors.

[6] Hung N.B., Lim O.T. (2019). A simulation and experimental study on the operating characteristics of a solenoid gas injector. Adv. Mech. Eng..

[7] Czarnigowski J. (2014). Experiments on the Effect of Pressure and Voltage Supply on Pulse Injector Opening Time. SAE Tech. Pap..

[8] Czarnigowski J., Jakliński P., Zyska T. (2014). An empirical model of current in the pulse gas injector’s circuit. Prz. Elektrotech..

[9] Chai B., Gao W. Simulation on fuel injection system for EUP based on AMESim. Proceedings of the 2010 Second International Conference on Computer Modeling and Simulation.

[10] Mitukiewicz G., Burdzik R., Leyko J. (2015). Relationship between LPG fuel and gasoline injection duration for gasoline direct injection engines. Fuel.

[11] Rozowicz S. Modelling the ignition system of combustion engines with two-port network. Proceedings of the 2017 18th International Symposium on Electromagnetic Fields in Mechatronics, Electrical and Electronic Engineering.

[12] Kubis M., Beno P. (2019). Realization of communication via the CAN bus. Transp. Res. Procedia.

[13] Fan Q., Bian J., Lu H., Tong S., Li L. (2012). Misfire detection and re-ignition control by ion current signal feedback during cold start in two-stage direct-injection engines. Int. J. Engine Res..

[14] Traistaru A., Sora I. (2010). Real Time Estimation of Li-Ion Battery Resistance Used in the Automotive Industry. J. Electr. Eng..

[15] Xiao L., Zhang Z., Guo H. (2010). Research on the Opening and Closing Times of an Electromagnetic Injector. J. Univ. Shanghai Sci. Technol..

[16] Ma Z., Qian Y., Yu X. (1997). Mathematical Model for the Injection Process of the Electronic Controlled Injectors. Trans. CSICE.

[17] Yin C., Zhang Z., Liu Z. (2007). Experimental Research and Design for Electronic Control Injector Test about Flow Characteristic. J. Agric. Mech. Res..

[18] Chen L., Zhang Z. Study on the measurement of dynamic characteristics for automotive electronic fuel injector. Proceedings of the 2011 International Conference on Transportation, Mechanical, and Electrical Engineering (TMEE).

[19] Wieclawski K., Antkowiak M., Figlus T. (2022). Recognizing Significant Components of Electrical Waveforms of Actuators Operated by Vehicle Controllers. Sensors.

